# Bidirectional Association Between Psoriasis and Obstructive Sleep Apnea: A Systematic Review and Meta-Analysis

**DOI:** 10.1038/s41598-020-62834-x

**Published:** 2020-04-03

**Authors:** Tzong-Yun Ger, Yun Fu, Ching-Chi Chi

**Affiliations:** 1 Department of Dermatology, Chang Gung Memorial Hospital, Linkou, Taoyuan Taiwan; 2 College of Medicine, Chang Gung University, Taoyuan, Taiwan

**Keywords:** Risk factors, Skin diseases

## Abstract

The link between psoriasis and obstructive sleep apnea (OSA) has not been confirmed. We aimed to investigate the relationship between psoriasis and OSA. We conducted a systematic review and meta-analysis of case-control, cross-sectional, and cohort studies on the association between psoriasis and OSA. We searched MEDLINE and Embase for relevant studies on May 11, 2019. The Newcastle-Ottawa Scale was used to evaluate the risk of bias of included studies. We performed random-effects model meta-analysis to calculate pooled odds ratio (ORs) with 95% confidence intervals (CIs) for case-control and cross-sectional studies as well as pooled incidence rate ratio (IRR) with 95% CIs for cohort studies in association between psoriasis and OSA. A total of 4 case-control or cross-sectional studies and 3 cohort studies with a total of 5,840,495 subjects were included. We identified a significantly increased odds for OSA in psoriasis patients (pooled OR 2.60; 95% CI 1.07–6.32), and significantly increased risk for psoriasis in OSA patients (pooled IRR 2.52; 95% CI 1.89–3.36). In conclusion, our study identified a bidirectional association between psoriasis and OSA. Sleep quality should be inquired in patients with psoriasis. Respirologist consultation or polysomnography may be indicated for those presenting with night snoring, recurrent awaking, and excessive daytime sleepiness.

## Introduction

Psoriasis is a chronic inflammatory skin disease with characteristic feature of sharply circumscribed erythematous plaques with silvery scales on the trunk and limbs^1^. The prevalence was estimated 0.5–11.4% in adults^2^. Psoriasis is considered as a multisystemic disease involving proinflammatory cytokines such as tumor necrosis factor (TNF), interleukin (IL)-1, and IL-17^3,4^. Various comorbidities have been linked to psoriasis including cardiovascular disease, metabolic syndrome, chronic kidney disease, uveitis, thyroid diseases, vitiligo, and inflammatory bowel disease^5–11^.

Obstructive sleep apnea (OSA), also called obstructive sleep apnea-hypopnea syndrome, is a chronic disorder of intermittent upper airway collapse during sleep resulting in recurrent hypoxia^12,13^. OSA is characterized with night snoring, recurrent awaking, and excessive daytime sleepiness^14^. The gold standard test for diagnosing OSA is polysomnography^15^. The prevalence of OSA ranges from 3 to 17%^12^. Risk factors of OSA include obesity, aging, male gender, anatomical predisposition, and alcohol consumption^12,13^. OSA induces systemic inflammatory and increases the risk of hypertension, stroke, cardiovascular disease, and metabolic disorder, in particular of diabetes mellitus type 2 and metabolic syndrome regardless of the obesity^15–17^.

Psoriasis and OSA share a common pathogenesis of inflammatory and immune imbalance^18,19^. The association between psoriasis and OSA has been examined in many studies but the results were limited and inconsistent^20–27^. The objective of this study was to assess the evidence regarding the bidirectional association between psoriasis and OSA.

## Methods

We conducted a systematic review and meta-analysis of observational studies (case-control, cross-sectional, and cohort studies) on the association between psoriasis and OSA. The reporting of this study followed the Meta-analysis of Observational studies in Epidemiology (MOOSE) guidelines^28^. We have registered the study protocol with PROSPERO (CRD42019129605; see https://www.crd.york.ac.uk/PROSPERO/display_record.php?RecordID=129605).

### Literatures search

We searched the MEDLINE and Embase for relevant studies from inception to May 11, 2019. The search terms included psoriasis and sleep apnea and their synonyms. The search strategy is shown in the Supplement. No geographic or language limitations were imposed.

### Study selection

The criteria for studies to be included were as follows: (1) observational studies investigating the association between psoriasis and OSA, including case-control, cross-sectional, and cohort studies; (2) research on human subjects; (3) the case group consisted of patients with psoriasis and the control group consisted of people without psoriasis in studies that examined the odds or risk of OSA in psoriasis patients; the study group was composed of patients with OSA and the control group was composed of people without OSA in studies that evaluated the odds or risk of psoriasis in OSA patients. We screened titles and abstracts for the initial study selection and obtained the full text of potentially eligible studies to confirm if they met our inclusion criteria. Two authors (T.G. and Y.F.) independently selected studies and disagreement was resolved by consulting the other author (C.C.).

### Data extraction

We extracted the following data from the included studies: first author, year of publication, country of study, number and gender of study subjects, definition of the case group, definition of outcome, and risk estimates including hazard ratio (HR), incidence rate ratio (IRR), and odds ratio (OR) with 95% confidence interval (CI) on the association between psoriasis and OSA.

### Risk of bias evaluation

The risk of bias of included studies was assessed by using the Newcastle-Ottawa Scale^29^. For case-control and cross-sectional studies, we examined the following eight domains: adequacy of case definition, representativeness of cases, selection of controls, definitions of controls, comparability of cases and controls, ascertainment of exposure, same method of ascertainment for cases and controls, and non-response rate. For cohort studies, we evaluated the following eight domains: representativeness of exposed cohort, selection of non-exposed cohort, ascertainment of exposure, absence of outcome of interest at start of study, comparability of cohorts, assessment of outcome, follow-up duration, and adequacy of follow up of cohorts.

### Statistical analysis

The Review Manage version 5.3 (Copenhagen: The Nordic Cochrane Centre, The Cochrane Collaboration, 2014) was used for conducting all analyses. We calculated the OR with 95% CI in included case-control and cross-sectional studies and IRR in included cohort studies. If OR was not reported in an included study, we calculated the crude OR based on published data for example the number of events in the case and control groups. The most fully adjusted OR, HR, or IRR were adopted if reported. We performed meta-analyses to evaluate the bidirectional association between psoriasis and OSA. For included case-control and cross-sectional studies, we calculated pooled OR to examine the association between the two diseases. For cohort studies, we treated HR as IRR and calculated the pooled IRR^30^. The statistical heterogeneity was assessed by the I^2^ statistic across the included studies. An I^2^ of >50% represents substantial heterogeneity^31^. We chose random-effects model for meta-analyses because clinical heterogeneity was anticipated. Therefore the DerSimonian and Laird method that takes between-study variability into account was used to obtain the pooled OR and IRR estimates^32^. Also, we conducted a sensitivity analysis after excluding studies that were rated with a high risk of bias.

## Results

### Characteristic of included studies

**Figure 1:**
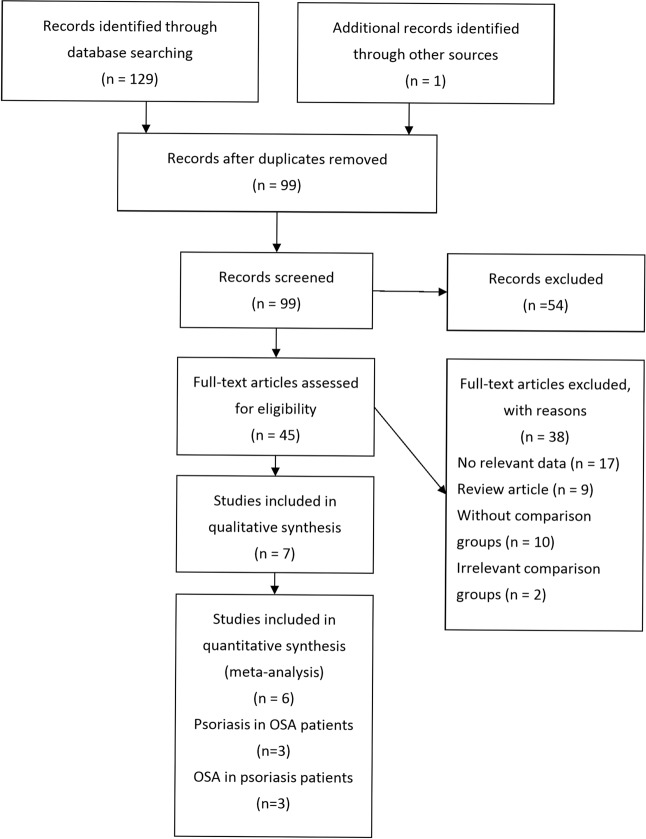
PRISMA study flow diagram.

The PRISMA flow chart of study selection is shown in Fig. 1. Our systematic literature search yielded 98 records after removing duplicates. We identified one additional relevant study in a review article. After screening the titles and abstracts, 54 records were excluded. After assessing the full text for eligibility, 38 articles were excluded due to no relevant data, review articles, no comparison group or irrelevant comparison group. Ultimately seven studies with a total of 5,840,495 study subjects were included. One cohort study, two case control studies, and one cross-sectional study investigated the association of psoriasis with OSA. Three cohort studies and one case-control study investigated the association of OSA with psoriasis. There was one cohort study that reported the bidirectional association between psoriasis and OSA. The main characteristics of included studies are summarized respectively in Table 1.

**Table 1:** Characteristics of included studies.

First author, year, country	Study design	Case group	Control group	Case definition and sampling population/outcome definition	Results	
**Studies investigating the odds of obstructive sleep apnea in psoriasis patients**						
Tsai, 2011, Taiwan	Case-control	51,800 patients with psoriasis (31,923 males and 19,877 females)	207,200 age-, gender- and urbanization- matched controls (127,692 males and 79,508 females)	ICD-9-CM psoriasis code from national health insurance database in 2006/ICD-9-CM sleep apnea code	Crude OR: 3.30 (2.00–5.44)	Adjusted RR: 3.89 (2.26–6.71)
Shalom, 2016, Israel	Case-control	12,336 patients with psoriasis (6,441 males and 5,895 females)	24,008 age- and sex- matched controls (12,096 males and 11,912 females)	Diagnosis of psoriasis by dermatologists from medical database of Clalit Health Services/ICD-9-CM sleep apnea code	OR:1.74 (1.50–2.03)	Adjusted OR: 1.27 (1.08–1.49)
Egeberg, 2016, Denmark	Cohort study	66,523 patients with psoriasis (32,115 males and 34,408 females)	5,393,040 individuals in the reference population (2,659,620 males and 2,733,420 females)	ICD-10-CM psoriasis or psoriatic arthritis code from Danish National Patient Register from 1 Jan 1997 to 31 Dec 2011/ICD-10-CM sleep apnea code	IRR: Mild psoriasis: 1.88 (1.69–2.09) Severe psoriasis: 2.69 (2.00–3.62) Psoriatic arthritis: 3.08 (2.38–4.00)	Adjusted IRR: Mild psoriasis: 1.36 (1.21–1.53) Severe psoriasis: 1.53 (1.08–2.18) Psoriatic arthritis: 1.98 (1.50–2.61)
Sacmaci, 2019, Turkey	Cross-sectional	60 patients with psoriasis (30 males and 30 females)	60 sex- and age- matched controls (30 males and 30 females)	Diagnosis of psoriasis by dermatologists//Diagnosis of sleep apnea by neurologists according to Berlin Questionnaire for Sleep Apnea	Crude OR: 6 (1.89–19.04)	NA
**Studies investigating the odds of psoriasis in obstructive sleep apnea patients**					**Crude (95% CI)**	**Adjusted** **(95% CI)**
Yang, 2012, Taiwan	Cohort study	2,258 patients with sleep apnea (1,414 males and 844 females)	11,255 age- and sex-matched controls without sleep apnea and psoriasis	ICD-9-CM sleep apnea codes from national health insurance database from 1 Jan 2001 to 31 Dec 2005 after receiving polysomnography/Two consensus psoriasis diagnosis, with at least one made by dermatologist or rheumatologists	Crude HR: 2.21 (1.08–4.49)	Adjusted HR: 2.30(1.13–4.69)
Cohen, 2015, USA	Cohort study	490 patients with OSA (All females)	71,108 individuals in the reference population (All females)	Self-reported sleep apnea in 1997/Self-reported psoriasis, psoriasis within 2 years of onset of sleep apnea were excluded	Crude IRR: 2.65 (1.6–4.14)	Adjusted RR: 1.91(1.20–3.05)
Egeberg, 2016, Denmark	Cohort study	39,908 patients with sleep apnea (31503 males and 8405 females)	5,419,655 individuals in the reference population (2,660,232 males and 2,759,423 females)	ICD-10-CM sleep apnea code from Danish National Patient Register from 1 Jan 1997 to 31 Dec 2011/ICD-10-CM psoriasis or psoriatic arthritis code	IRR: Sleep apnea without CPAP Mild psoriasis: 1.97 (1.71–2.26) Severe psoriasis: 2,80 (2.02–3.87) Psoriatic arthritis: 2.38 (1.65–3.34) Sleep apnea with CPAP Mild psoriasis: 2.29 (1.79–2.93) Severe psoriasis: 4.77 (2.96–7.67) Psoriatic arthritis: 7.29 (4.88–10.88)	Adjusted IRR: Sleep apnea without CPAP Mild psoriasis: 1.62 (1.38–1.89) Severe psoriasis: 1.85 (1.25–2.74) Psoriatic arthritis: 1.98 (1.32–2.99) Sleep apnea with CPAP Mild psoriasis: 1.95 (1.48–2.57) Severe psoriasis: 3.75 (2.22–6.34) Psoriatic arthritis: 6.84 (4.49–10.4)
Papadavid, 2017, Greece	Case-control	253 patients with OSA (200 males and 53 females)	104 controls without OSA (82 males and 22 females)	Underwent full nocturnal polysomnography, cessation of airflow for ≥10 s, from July 2009 to July 2012/Diagnosed for psoriasis by the same dermatologist	NA	Adjusted OR: 13.31 (1.19–48.93)

CI, confidence interval; CM, Clinical Modification; HR, hazard ratio; ICD, International Classification of Disease; IRR, incidence rate ratio; N/A, not available; OR, odds ratio; OSA, obstructive sleep apnea; RR, risk ratio.

### Risk of bias of included studies

**Figure 2:**
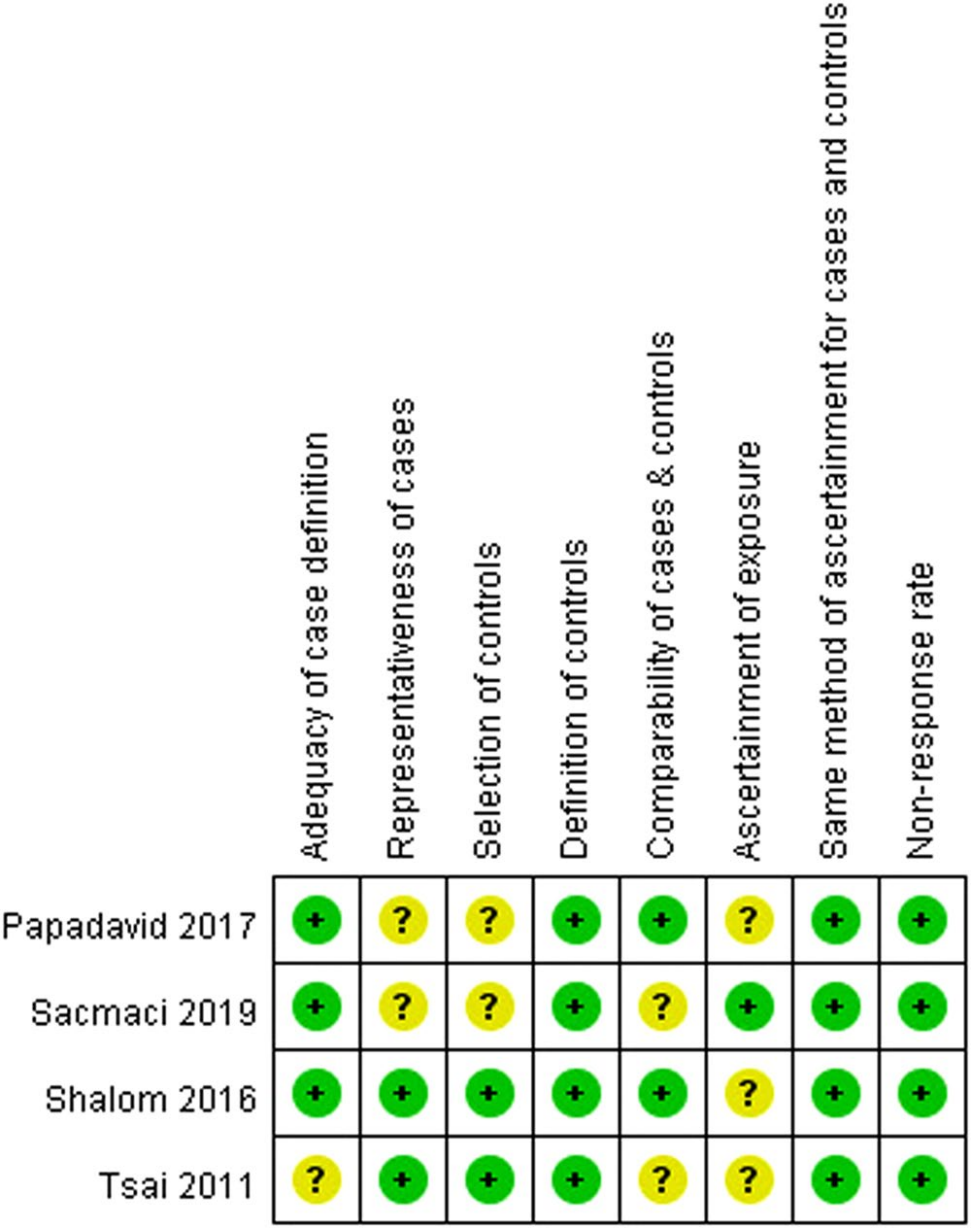
Risk of bias of included case-control and cross-sectional studies. Risk of bias were assessed base on Newcastle-Ottawa Scale. Green dots indicate low risk of bias; yellow dots indicate unclear risk of bias and red dots indicate high risk of bias.

**Figure 3:**
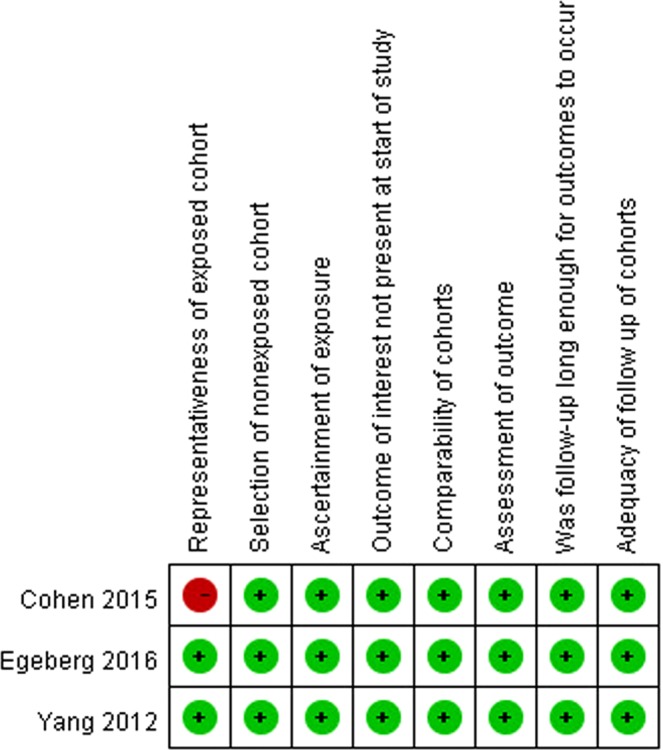
Risk of bias of included cohort studies. Risk of bias were assessed base on Newcastle-Ottawa Scale. Green dots indicate low risk of bias; yellow dots indicate unclear risk of bias and red dots indicate high risk of bias.

The risk of bias of included studies was summarized in Figs. 2 and 3. Of four included case-control and cross-sectional studies, no items were rated as high risk of bias (see Fig. 2). The Tsai 2011 study was rated with an unclear risk of bias in the ‘adequacy of case definition’ domain because only the International Classification of Disease (ICD) diagnostic codes were used to identify patients^33^. Two studies were rated with an unclear risk of bias in the ‘representativeness of cases’ and ‘selection of controls’ domains because patients were selected from hospital not community^34,35^. We rated two studies as unclear risk of bias in the ‘comparability of cases and controls’ domain because only age and gender, but not body mass index, were controlled^33,35^. Moreover, three studies were rated as unclear risk in the ‘ascertainment of exposure’ domain because only medical records or ICD codes were employed^24,33,34^. In three included cohort studies, we rated the Cohen 2015 study at high risk of bias in the ‘representativeness of exposed cohort’ domain (see Fig. 3)^23^. The reason was that the study subjects were all female nurses.

### Bidirectional association between OSA and psoriasis

**Figure 4:**
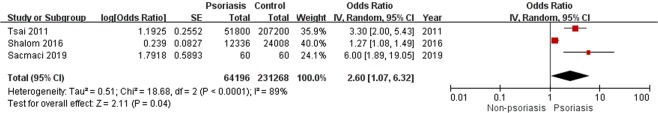
Forest plot for case-control and cross-sectional studies on the association of psoriasis with obstructive sleep apnea. The meta-analysis illustrated a significant association of obstructive sleep apnea with psoriasis (pooled odds ratio 2.60; 95% confidence interval 1.07–6.32).

One cohort study^25^, one cross-sectional study^35^, and two case-control studies^24,33^ demonstrated an increase in OSA among patients with psoriasis. The meta-analysis of two included case-control and one cross-sectional studies with 295,464 study subjects showed a significant association of psoriasis with OSA (pooled OR, 2.60; 95% CI, 1.07–6.32; Fig. 4)^24,33,35^. Considerable statistical heterogeneity was found (I^2^ = 89%; P < 0.0001). One included cohort study illustrated a consistently increased risk for OSA in patients with mild psoriasis (adjusted IRR 1.36; 95% CI 1.21–1.53), severe psoriasis (adjusted IRR 1.53; 95% CI 1.08–2.18), and psoriatic arthritis (adjusted IRR 1.98; 95% CI 1.50–2.61)^25^.

**Figure 5:**
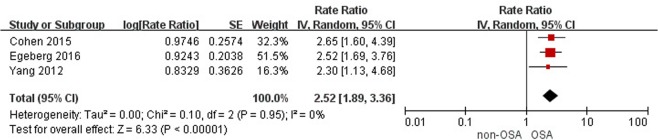
Forest plot for cohort studies on the association of obstructive sleep apnea with psoriasis. The meta-analysis illustrated a significant association of obstructive sleep apnea with psoriasis (pooled incidence rate ratio 2.52; 95% confidence interval 1.89–3.36).

Conversely, three cohort studies^23,25,27^ and one case-control study^34^ found a consistent increase in psoriasis among patients with OSA. The meta-analysis of three included cohort studies with 5,544,674 study subjects showed a significant association of OSA with psoriasis (pooled IRR, 2.52; 95% CI, 1.89–3.36; Fig. 5)^23,25,27^. There was no statistical heterogeneity within these studies (I^2^ = 0%). The Cohen 2015 study was rated with a high risk of bias in the representativeness of the exposed cohort because all the study subjects were nurses^23^. However, the association of OSA with psoriasis remained positive after excluding the Cohen 2015 study (pooled IRR 2.47; 95% CI 1.74–3.49). One case-control study showed a significantly increased odds for psoriasis in relation to OSA (adjusted OR 13.31, 95% CI 1.19–48.93)^34^.

## Discussion

To the best of our knowledge, the present study is the first meta-analysis investigating the bidirectional association between psoriasis and OSA. The evidence from included case-control and cross-sectional studies found a 2.6-fold greater odds for prevalent OSA in relation to psoriasis^24,33,35^ and a 13-fold increased odds for prevalent psoriasis in relation to OSA^34^. Similarly, the evidence from an included cohort study demonstrated consistently increased risk for incident OSA among patients with mild and severe psoriasis as well as psoriatic arthritis^25^. Conversely, OSA patients were 2.52-fold more likely to develop incident psoriasis when compared with non-OSA controls^23,25,27^. Our meta-analysis possesses high generalizability because of including studies from various ethnicities for example the United States, France, Denmark, Greece, Israel, and Taiwan.

Obesity is a trigger for inflammation and has been linked to chronic inflammatory diseases^36^. Patients with psoriasis have been found to have increased body mass index (BMI) than healthy controls^37^. Besides, OSA was related to obesity and thus weight loss is the recommended fist-line therapy^38,39^. Obesity may promote upper airway inflammation, reduce muscle contractibility, and induce airway collapse during sleep^40^. BMI has been correlated with psoriasis severity^37^ and OSA^26^. Among most of included studies in our meta-analysis^23–25,27,34^, the confounding from obesity has been considered and BMI was adjusted in the statistical analysis. The bidirectional association between psoriasis and OSA remained significant after adjustment for obesity or BMI. Only one included case-control study of Tsai 2011 did not contain any information about BMI or body weight^33^. Another included cross-sectional study of Sacmaci 2019 did not perform adjustment for obesity, but there was no significant difference in BMI between psoriasis patients and non-psoriasis controls (28.5 kg/m^2^ vs. 26.9 kg/m^2^; P = 0.051)^35^. Therefore, confounding by obesity could not fully explain the association between psoriasis and OSA.

The bidirectional association between psoriasis and OSA suggest the shared common systemic inflammatory pathogenic pathways^19^. OSA is considered a systemic inflammatory disorder. High levels of systemic inflammatory molecules are part of mechanism leading to OSA^41,42^. The activities of IL-17, TNF, IL-6, IL-7 and C-reactive protein are significantly increased in patients with OSA compare to obese patients^43–45^. These pro-inflammatory cytokines decreased after continuous positive airway pressure (CPAP) treatment^44^. Studies have shown that the circadian rhythm of TNF release was significantly disturbed in patients with OSA^46,47^. The inflammatory process may predispose them to the development of psoriasis^27^. Elevated circulating levels of IL-17, TNF, IL-6, and IL-22 were also associated with psoriasis^48^. One previous study found that treatment with etanercept significantly improved symptoms of OSA in moderate to severe psoriasis^49^. The increase of IL-17 is related to atherosclerotic vascular disease, which is a risk factor for OSA^50^. Moreover, psoriasis causes pruritus and may lead to sleep disturbance, which contributes to systemic inflammation and consequently induces OSA^51–53^.

Autonomic activation is considered an important factor between OSA and psoriasis^53–55^. Some scholars assumed that psoriasis-related itching and pain could disrupt sleep and thus increase autonomic activation, which possibly induces OSA^18,54^. On the other hand, Gabryelska *et al*. proposed low grade inflammation in OSA patients with elevated levels of IL-1 and TNF could stimulate hypothalamus and increase hypothalamic-pituitary-adrenal activity with resultant surging of autonomic activity^56^. The inflammation and autonomic activation involved in OSA may be a risk factor for psoriasis^55^.

Intermittent hypoxia during sleep in OSA patients induces oxidative stress. Some markers including nuclear factor-κB (NF-κB) and hypoxia-inducible factor-1α (HIF-1α) could detect the oxygen stress and activate the inflammatory pathway^57^. HIF-1α, a transcriptional regulator of cell oxygen metabolism, is a specific marker for diagnosing OSA^58^. The levels of HIF-1α correlate with the severity of OSA and can be effectively reduced by CPAP treatment^57^. Besides, vascular risk response to hypoxia was detected in OSA patients. A significant upregulation of NF-κB, HIF-1α, endothelial nitric oxide synthase, vascular cell adhesion molecule 1, and vascular endothelial growth factor (VEGF) were expressed in the skin biopsy specimens of OSA patients^59^. Increased circulation of VEGF promotes angiogenesis which is also an important process in initiating psoriasis^59,60^. VEGF is a marker of tissue response to hypoxia and activated by HIF-1α^59^. There is evidence indicating activation of excess HIF-1α and VEGF in psoriatic skin when compare to normal skin^61,62^. In addition, HIF-1α is involved in T-cell regulation and survival which are crucial in psoriasis formation^62^. The effects of hypoxia with elevated levels of HIF-1α and VEGF may explain the association between OSA and psoriasis.

There are a few limitations of the present study. First, OSA was identified by the use of ICD codes in four included studies^24,25,27,33^ and misclassification bias might have been present. Second, high statistical heterogeneity was detected as to the association of psoriasis with OSA (I^2^ = 89%; see Fig. 4), but all the include studies consistently reported positive association.

In conclusion, the evidence to date supports a bidirectional association between psoriasis and OSA. All patients with psoriasis should be informed about the risk of OSA, and vice versa. OSA is a comorbidity of psoriasis that should not be ignored, and sleep quality should be inquired in patients with psoriasis. Respirologist consultation or polysomnography may be indicated for those presenting with night snoring, daytime sleepiness, and insomnia.

## Supplementary information

Supplementary Table S1.

## References

[1] Nestle FO, Kaplan DH, Barker J (2009). Psoriasis. N. Engl. J. Med..

[2] Michalek IM, Loring B, John SM (2017). A systematic review of worldwide epidemiology of psoriasis. J. Eur. Acad. Dermatol. Venereol..

[3] Lowes MA, Bowcock AM, Krueger JG (2007). Pathogenesis and therapy of psoriasis. Nature.

[4] Griffiths CE (2006). Psoriasis and psoriatic arthritis: immunological aspects and therapeutic guidelines. Clin. Exp. Rheumatol..

[5] Machado-Pinto, J., Diniz Mdos, S. & Bavoso, N. C. Psoriasis: new comorbidities. *An. Brasileiros de. Dermatologia* **91**, 8–14, 10.1590/abd1806-4841.20164169 (2016). 10.1590/abd1806-4841.20164169 PMC4782640 26982772

[6] Malkic Salihbegovic E, Hadzigrahic N, Cickusic AJ (2015). Psoriasis and metabolic syndrome. Med. Arch..

[7] Chi CC (2015). Risk of incident chronic kidney disease and end-stage renal disease in patients with psoriasis: A nationwide population-based cohort study. J. Dermatol. Sci..

[8] Chi, C. C. *et al*. Risk of uveitis among people with psoriasis: A nationwide cohort study. *JAMA Ophthalmol.* **135**, 415–422, 10.1001/jamaophthalmol.2017.0569 (2017). 10.1001/jamaophthalmol.2017.0569 PMC5846892 28418500

[9] Wang SH, Wang J, Lin YS, Tung TH, Chi CC (2019). Increased risk for incident thyroid diseases in people with psoriatic disease: A cohort study. J. Am. Acad. Dermatology.

[10] Yen, H. & Chi, C. C. Association between psoriasis and vitiligo: a systematic review and meta-analysis. *Am. J. Clin. Dermatology* **20**, 31–40, 10.1007/s40257-018-0394-1 (2019). 10.1007/s40257-018-0394-1 30317450

[11] Fu Y, Lee CH, Chi CC (2018). Association of psoriasis with inflammatory bowel disease: a systematic review and meta-analysis. JAMA Dermatol..

[12] Badran M, Ayas N, Laher I (2014). Insights into obstructive sleep apnea research. Sleep. Med..

[13] Punjabi NM (2008). The epidemiology of adult obstructive sleep apnea. Proc. Am. Thorac. Soc..

[14] Kabeloglu Ilbay, V., Tas, B., Altuntas, M., Atakli, H. D. & Soysal, A. Risk of obstructive sleep apnea syndrome in psoriasis patients. *Arch. Iran. Med.* **22**, 137–143 (2019). 31029070

[15] Pagel JF (2007). Obstructive sleep apnea (OSA) in primary care: evidence-based practice. J. Am. Board. Family Medicine: JABFM.

[16] Park JG, Ramar K, Olson EJ (2011). Updates on definition, consequences, and management of obstructive sleep apnea. Mayo Clin. Proc..

[17] Bagai K (2010). Obstructive sleep apnea, stroke, and cardiovascular diseases. Neurologist.

[18] Hirotsu C (2015). The bidirectional interactions between psoriasis and obstructive sleep apnea. Int. J. Dermatology.

[19] Gupta MA, Simpson FC, Gupta AK (2016). Bidirectional relationship between obstructive sleep apnea (OSA) and psoriasis: Implications for OSA therapies?. J. Clin. Sleep. Med..

[20] Karaca S (2013). Might psoriasis be a risk factor for obstructive sleep apnea syndrome?. Sleep. Breath..

[21] Buslau M, Benotmane K (1999). Cardiovascular complications of psoriasis: does obstructive sleep apnoea play a role?. Acta Dermato-Venereologica.

[22] Dalamaga M, Papadavid E, Vlami K (2013). Unmasking the Janus face of the association between psoriasis, metabolic syndrome and obstructive sleep apnea. Sleep Breath..

[23] Cohen, J. M., Jackson, C. L., Li, T. Y., Wu, S. & Qureshi, A. A. Sleep disordered breathing and the risk of psoriasis among US women. *Arch. Dermatol. Res.* **307**, 433–438, 10.1007/s00403-015-1536-4 (2015). 10.1007/s00403-015-1536-4 25676527

[24] Shalom G, Dreiher J, Cohen A (2016). Psoriasis and obstructive sleep apnea. Int. J. Dermatol..

[25] Egeberg A (2016). Psoriasis and sleep apnea: A Danish nationwide cohort study. J. Clin. Sleep. Med..

[26] Papadavid E (2013). Sleep apnea as a comorbidity in obese psoriasis patients: a cross-sectional study. Do psoriasis characteristics and metabolic parameters play a role?. J. Eur. Acad. Dermatol. Venereol..

[27] Yang YW, Kang JH, Lin HC (2012). Increased risk of psoriasis following obstructive sleep apnea: A longitudinal population-based study. Sleep Med..

[28] Stroup DF (2000). Meta-analysis of observational studies in epidemiology: a proposal for reporting. Meta-analysis Of Observational Studies in Epidemiology (MOOSE) group. JAMA.

[29] Wells, G., Shea, B., O'Connell, J. *et al.* *The Newcastle-Ottawa Scale (NOS) for assessing the quality of nonrandomised studies in meta-analyses*. http://www.ohri.ca/programs/clinical_epidemiology/oxford.asp (2014).

[30] Hernán MA (2010). The hazards of hazard ratios. Epidemiology.

[31] Higgins JP, Thompson SG (2002). Quantifying heterogeneity in a meta-analysis. Stat. Med..

[32] DerSimonian R, Laird N (1986). Meta-analysis in clinical trials. Controlled Clin. trials.

[33] Tsai TF (2011). Epidemiology and comorbidities of psoriasis patients in a national database in Taiwan. J. Dermatol. Sci..

[34] Papadavid E (2017). Psoriasis is associated with risk of obstructive sleep apnea independently from metabolic parameters and other comorbidities: a large hospital-based case-control study. Sleep. Breath..

[35] Saçmacı, H. & Gürel, G. Sleep disorders in patients with psoriasis: a cross-sectional study using non-polysomnographical methods. *Sleep Breath.*, 10.1007/s11325-019-01820-8 (2019). 10.1007/s11325-019-01820-8 30859369

[36] Versini M, Jeandel PY, Rosenthal E, Shoenfeld Y (2014). Obesity in autoimmune diseases: not a passive bystander. Autoimmunity Rev..

[37] Duarte GV (2013). Association between obesity measured by different parameters and severity of psoriasis. Int. J. Dermatol..

[38] Strobel RJ, Rosen RC (1996). Obesity and weight loss in obstructive sleep apnea: a critical review. Sleep.

[39] Foster GD (2009). A randomized study on the effect of weight loss on obstructive sleep apnea among obese patients with type 2 diabetes: the Sleep AHEAD study. Arch. Intern. Med..

[40] Vgontzas AN (2008). Does obesity play a major role in the pathogenesis of sleep apnoea and its associated manifestations via inflammation, visceral adiposity, and insulin resistance?. Arch. Physiol. Biochem..

[41] Entzian P, Linnemann K, Schlaak M, Zabel P (1996). Obstructive sleep apnea syndrome and circadian rhythms of hormones and cytokines. Am. J. Respir. Crit. Care Med..

[42] Carpagnano GE (2002). Increased 8-isoprostane and interleukin-6 in breath condensate of obstructive sleep apnea patients. Chest.

[43] Ryan S, Taylor CT, McNicholas WT (2005). Selective activation of inflammatory pathways by intermittent hypoxia in obstructive sleep apnea syndrome. Circulation.

[44] Minoguchi K (2004). Elevated production of tumor necrosis factor-alpha by monocytes in patients with obstructive sleep apnea syndrome. Chest.

[45] Yue HJ (2009). The roles of TNF-alpha and the soluble TNF receptor I on sleep architecture in OSA. Sleep Breath..

[46] Strober, B. E. *et al*. Sleep quality and other patient-reported outcomes improve after patients with psoriasis with suboptimal response to other systemic therapies are switched to adalimumab: results from PROGRESS, an open-label Phase IIIB trial. *Br. J. Dermatol.* **167**, 1374–1381, 10.1111/bjd.12000 (2012). 10.1111/bjd.12000 22897348

[47] Sacmaci H, Gurel G (2019). Sleep disorders in patients with psoriasis: a cross-sectional study using non-polysomnographical methods. Sleep Breath..

[48] Arican O, Aral M, Sasmaz S, Ciragil P (2005). Serum levels of TNF-alpha, IFN-gamma, IL-6, IL-8, IL-12, IL-17, and IL-18 in patients with active psoriasis and correlation with disease severity. Mediators Inflamm..

[49] Thaçi, D. *et al*. Improvement in aspects of sleep with etanercept and optional adjunctive topical therapy in patients with moderate-to-severe psoriasis: results from the PRISTINE trial. *J. Eur. Acad. Dermatol. Venereol.* **28**, 900–906, 10.1111/jdv.12207 (2014). 10.1111/jdv.12207 23848989

[50] Hashmi S, Zeng QT (2006). Role of interleukin-17 and interleukin-17-induced cytokines interleukin-6 and interleukin-8 in unstable coronary artery disease. Coron. Artery Dis..

[51] Shutty, B. G. *et al*. Sleep disturbances in psoriasis. *Dermatology Online J.* **19**, 1 (2013). 23374943

[52] Yosipovitch, G., Goon, A., Wee, J., Chan, Y. H. & Goh, C. L. The prevalence and clinical characteristics of pruritus among patients with extensive psoriasis. *Br. J. Dermatol.* **143**, 969–973, 10.1046/j.1365-2133.2000.03829.x (2000). 10.1046/j.1365-2133.2000.03829.x 11069504

[53] Gabryelska A, Sochal M, Wasik B, Bialasiewicz P (2018). Patients with obstructive sleep apnea are over four times more likely to suffer from psoriasis than the general population. J. Clin. Sleep. Med..

[54] Gupta MA, Gupta AK (2018). Psoriasis is associated with a higher prevalence of obstructive sleep apnea and restless legs syndrome: A possible indication of autonomic activation in psoriasis. J. Clin. Sleep. Med..

[55] Gabryelska A, Bialasiewicz P (2018). Is autonomic activation a middleman between obstructive sleep apnea syndrome and psoriasis?. J. Clin. Sleep. Med..

[56] Spath-Schwalbe, E., Gofferje, M., Kern, W., Born, J. & Fehm, H. L. Sleep disruption alters nocturnal ACTH and cortisol secretory patterns. *Biol. Psychiatr.* **29**, 575–584, 10.1016/0006-3223(91)90093-2 (1991). 10.1016/0006-3223(91)90093-2 1647222

[57] Lu D, Li N, Yao X, Zhou L (2017). Potential inflammatory markers in obstructive sleep apnea-hypopnea syndrome. Bosn. J. basic. Med. Sci..

[58] Gabryelska, A. *et al*. Serum Hypoxia-Inducible Factor-1alpha protein level as a diagnostic marker of obstructive sleep apnea. *Pol. Arch. Intern. Med.*, 10.20452/pamw.15104 (2019). 10.20452/pamw.15104 31834288

[59] Kaczmarek E (2013). Molecular biomarkers of vascular dysfunction in obstructive sleep apnea. PLoS one.

[60] Marina ME (2015). VEGF involvement in psoriasis. Clujul Med..

[61] Detmar, M. Evidence for vascular endothelial growth factor (VEGF) as a modifier gene in psoriasis. *J. Investig. Dermatol.* **122**, xiv–xv, 10.1046/j.0022-202X.2003.22140.x (2004). 10.1046/j.0022-202X.2003.22140.x 14962120

[62] Rosenberger, C. *et al*. Upregulation of hypoxia-inducible factors in normal and psoriatic skin. *J. Invest. Dermatol.* **127**, 2445–2452, 10.1038/sj.jid.5700874 (2007). 10.1038/sj.jid.5700874 17495954

