# Supplementary material for: Bidirectional Association Between Psoriasis and Obstructive Sleep Apnea: A Systematic Review and Meta-Analysis
Source: Sci Rep. 2020 Apr 3;10:5931. doi: 10.1038/s41598-020-62834-x (PMC7125081; doi:10.1038/s41598-020-62834-x)
Supplement: Supplementary file 1 — Supplementary Table S1. [file 41598_2020_62834_MOESM1_ESM.docx]

**Supplementary Material**

**Bidirectional Association Between Psoriasis and** **Obstructive Sleep Apnea: A Systematic Review and Meta-Analysis**

Tzong-Yun Ger, MD^1^; Yun Fu, MD^1^; Ching-Chi Chi, MD, MMS, DPhil^1,2^

^1^Department of Dermatology, Chang Gung Memorial Hospital, Linkou, Taoyuan, Taiwan. ^2^College of Medicine, Chang Gung University, Taoyuan, Taiwan.

Correspondence and requests for materials should be addressed to C.-C. Chi (email: chingchi@cgmh.org.tw)

**Figure legends for supplementary figures**

**Supplementary Table S1:** Search Strategy

**Table S1.** Search Strategy

| **MEDLINE search strategy**   1. psoria$.ti,ab.   2. exp Sleep Apnea Syndromes/  3. exp Snoring/  4. (sleep adj3 (apnea or apnoea or hypopnea or hypopnoea)).ti,ab.  5. ('upper airway resistance' or 'sleep disordered breathing' or snor$).ti,ab.  6. or/2-5  7. 1 and 6 |
| --- |
| **Embase search strategy**   1. psoriasis:ti,ab 2. psoriatic:ti,ab 3. #1 OR #2 4. 'sleep disordered breathing'/exp 5. 'snoring'/exp 6. (sleep NEAR/3 (apnea OR apnoea OR hypopnea OR hypopnoea)):ti,ab 7. 'upper airway resistance':ti,ab OR 'sleep disordered breathing':ti,ab OR snor$:ti,ab 8. #4 OR #5 OR #6 OR #7 9. #3 AND #8 |
